# The Content and Nature of Rumination in Chinese Young and Middle-Aged Patients with Acute Coronary Syndrome: A Qualitative Study

**DOI:** 10.3390/healthcare12161651

**Published:** 2024-08-19

**Authors:** Anan Li, Siying Ji, Yangfan Nie, Meixuan Chi, Naijuan Wang, Zhaoying Zhu, Shan Li, Yunying Hou

**Affiliations:** 1 Department of Nursing, The First Affiliated Hospital of Soochow University, No.188, Shizi Street, Gusu District, Suzhou 215006, China; 20225231006@stu.suda.edu.cn (A.L.);; 2 School of Nursing, Suzhou Medical College of Soochow University, No.333, Ganjiang East Road, Gusu District, Suzhou 215006, China

**Keywords:** acute coronary syndrome, young and middle aged, rumination, post-traumatic growth, qualitative study

## Abstract

Individuals have different rumination patterns after experiencing traumatic events in different cultural backgrounds and situations. This study aimed to explore the experience of Chinese young and middle-aged patients with acute coronary syndrome (ACS) to understand the content and nature of their rumination. Sixteen participants were selected using the purposive sampling method in the First Affiliated Hospital of Soochow University from May 2023 to December 2023. Data were collected using semi-structured interviews and analyzed using Colaizzi’s seven-step phenomenological method. The participants went through two successive stages: non-adaptive rumination and adaptive rumination. During the initial admission phase, all the participants experienced varying degrees of non-adaptive rumination. Non-adaptive rumination included four sub-themes: worry or anxiety of daily activities and medical therapy (37.50%), fear of unpredictable outcomes and death (37.50%), sadness of maladjustment (25.00%), and remorse of carelessness (12.50%). During the period of stable condition and pre-discharge, the participants received health education and gradually all transitioned to adaptive rumination. Adaptive rumination included four sub-themes: tracing of disease processes (100%), enhancement of disease cognition (81.25%), improvement of health awareness (62.50%), and adjustment of lifestyle cognition (100%). In conclusion, although the Chinese young and middle-aged patients with ACS experienced negative emotions after a traumatic cardiac event, they gradually made positive changes, and optimism and information support played important roles in this transition. The results of this study provide a fundamental understanding of rumination experiences in Chinese young and middle-aged patients with ACS and provide new data for healthcare providers when designing intervention programs to enhance post-traumatic growth in these patients.

## 1. Introduction

Acute coronary syndrome (ACS) is an acute ischemic heart disease with high morbidity and mortality, caused by the rupture, ulceration, or erosion of atherosclerotic plaques in the coronary arteries [1]. Globally, the incidence of acute myocardial infarction (AMI), the main type of ACS, is increasing among the population aged 18–59 years, and the incidence of MI was 12.9, 38.2, and 71.2 per 1000 in men and 2.2, 5.2, and 13.0 per 1000 in women in the age groups of 30 to 34, 35 to 44, and 45 to 54 years, respectively [2]. In China, according to a relevant study, the number of patients with AMI is predicted to reach 75 million by 2030, with young and middle-aged patients accounting for about 50% [3].

As a traumatic cardiac event [4], ACS can trigger post-traumatic stress symptoms (PTSSs) [5], including intrusive thoughts, avoidance, and negative alterations in cognition [6]. About 12% of patients with ACS may experience clinically significant PTSSs, which have been demonstrated to double the risk of recurrence of a coronary event or of death due to ACS [7]. Furthermore, ACS could also cause emotional disorders, such as severe or mild depressive episodes, chronic depression, anxiety, and type A behavior, leading to poor quality of life and impaired function of patients [8,9]. A cross-sectional survey showed that the prevalence of depression and anxiety symptoms in patients with ACS was 66.3% and 56.5%, respectively [10]. The most common depressive symptoms of patients with ACS are the presence of sadness and emptiness, and the most common anxiety symptoms are excessive fear and anxiety. 

Although ACS is a stressful, challenging, and traumatic event, this difficult experience may promote personal growth for ACS survivors, known as post-traumatic growth (PTG) [11]. PTG refers to the positive changes that an individual makes in multiple aspects after struggling with a trauma, including an increased appreciation for life, more meaningful interpersonal relationships, an increased sense of personal strength, and a richer existential and spiritual life [12,13]. PTG has been shown to occur in individuals experiencing AMI. In the study by Norekvål et al., the majority of female patients with AMI (65%) reported positive effects from their AMI experience [14]. Zhou et al. reported moderate PTG levels in young and middle-aged patients with AMI [11]. Wang et al. conducted a potential profile analysis of PTG in young and middle-aged patients with AMI and showed that 18.91% belonged to the “Good growth group”, and 35.58% belonged to the “Excellent growth group” [3]. Patients who developed PTG were less likely to have recurrent AMIs [15] and depressive symptoms and even had enhanced psychological well-being [16].

In 2018, Tedeschi et al. proposed a revised model of PTG development [17], and rumination was the central tenet of this model. Rumination refers to the cognitive processing patterns of individuals after experiencing a traumatic event, including non-adaptive and adaptive rumination (or intrusive and deliberate rumination). Non-adaptive rumination occurs immediately after a traumatic event and leads to emotional distress such as anxiety, depression, and fear [18]. This type of rumination occurs unintentionally and causes individuals to spend most of their time on it. Adaptive rumination enables individuals to make sense of life events, re-establish their beliefs, process the emotions they have experienced, and develop coping skills [19].

Zhou et al. reported moderate levels of rumination in young and middle-aged patients with AMI [11]. Rumination is a predictor of PTSSs and PTG [20]. Non-adaptive rumination has been demonstrated to be associated with the negative effects of trauma, such as dissociation state [21], insomnia [22], and depression [23]. Trick et al. found rumination to be an independent predictor of depression in patients with ACS [24]. On the other hand, adaptive rumination is a positive predictor for PTG in young and middle-aged patients with AMI [3]. The study by Zhou et al. demonstrated that adaptive rumination was positively correlated with PTG and mediated the relationship between PTG and psychological adjustment in young and middle-aged patients with AMI [11]. Thus, an intervention to modify the type of rumination can be used to promote the development of PTG.

To better develop a “framework of actions” for including patients in future research designs and PTG interventions, many researchers have used qualitative methods to explore the content and forms of rumination in individuals who had experienced traumatic events. Norekvål conducted qualitative interviews with 18 Norwegian elderly female patients with AMI on the nature of the perceived positive effects of the disease and extracted four themes: appreciating life (55%), obtaining health care (42%), making lifestyle changes (36%), and taking more care of self and others (29%) [14]. However, rumination is influenced by age [25] and social culture [26]. Compared to older adults, young and middle-aged adults are often the core of the family and the main workforce of society. After the diagnosis of ACS, patients will live with the disease for life, so they will likely bear a heavy burden in many aspects, such as expensive medical expenses; physical limitations; psychosocial maladjustment; risk of premature death; disharmonious family relationships; and the conflict between the roles of patients, families, and workers [3,27]. In addition, younger adults are more likely to dwell on negative moods [25] and use maladaptive strategies to a greater extent in high- and moderate-intensity situations and in situations that elicit anxiety and sadness. And maladjusted young adults have higher levels of psychological distress [28]. The impact of sociocultural differences on rumination is even more complex. Socioculture affects one’s perceptions of change, which in turn affects the extent to which people attribute the act of rumination to motivation for change and to improve or self-doubt one’s ability, which in turn has different effects on mental health [26]. Therefore, individuals of different ages or those living in different sociocultural contexts may develop different rumination patterns from a traumatic experience. In China, the research on rumination is mostly conducted in patients with cancer, especially breast cancer. There have been no qualitative studies on rumination in young and middle-aged patients with ACS. Therefore, it is essential to explore the real experiences and feelings regarding ACS of young and middle-aged patients with ACS using qualitative research methods, which will help to understand the content and nature of their rumination, thereby providing new data for the development of future intervention programs to promote PTG in these patients.

## 2. Materials and Methods

### 2.1. Design

This study was a qualitative research and used the Colaizzi’s phenomenological method to analyze data [29]. The framework and reporting of this study followed the Qualitative Research Reporting Integrated Standards (COREQ) reporting guidelines to ensure that sufficient details regarding the methods of data collection, analysis, and interpretation were provided. This study was approved by the Ethics Committee of Soochow University (SUDA20220922H02).

### 2.2. Participants

The participants were selected using the purposive sampling method in the First Affiliated Hospital of Soochow University from May 2023 to December 2023. The inclusion criteria were (i) patients who were diagnosed with ACS (i.e., unstable angina, ST-elevated myocardial infarction, and non-ST-elevated myocardial infarction); (ii) patients who were 18–59 years old, with good cognition and communication skills; and (iii) patients who were in stable condition and volunteered to participate in this study. Patients with severe life-threatening cardiac disease (e.g., cardiogenic shock, acute heart failure, and cardiac rupture), severe dysfunction of other organs, a history of psychiatric and psychological disorders, or malignant tumors were excluded. The interviewers selected potential participants from the medical system and subsequently had a brief conversation with them to ensure they met the inclusion and exclusion criteria for this study. If the potential participant met the inclusion and exclusion criteria for this study, the interviewer would invite them to join. One by one, the participants were recruited and interviewed until sample size adequacy was reached (i.e., information saturation occurred, and no new themes were identified). In total, 16 participants were included in this study to reach sample size adequacy, with no dropouts or refusals.

### 2.3. Interview Guide

The interview questions were designed based on the relevant literature and expert opinions. Pre-interviews were conducted with two patients to determine the interview guide. The interview guide is displayed in Supplementary Materials.

### 2.4. Interview

We conducted one-on-one, face-to-face semi-structured interviews. All the interviews were conducted in a private room to maintain the participants’ privacy and psychological security. Before the start of an interview, all the participants were informed of this study’s objectives and the voluntary nature of participation. Each participant signed a paper-based informed consent form and allowed audio recording of the interviews. Participants were assured of the confidentiality and anonymity of the recordings, transcripts, and any behaviors observed during the interviews. The participants were asked to provide demographic and clinical information (e.g., name, age, gender, educational level, occupation, and comorbidity). In order to ensure the privacy of the participants, numbers were used instead of their real names. Afterward, recording began, and the interviewer began to make small talk with the participant to make them relax and asked them the interview questions without them realizing it. During each interview, the interviewer listened to the participant’s answers, observed their movements and expression changes, and made records. According to the interview guide and the actual situation of participants, the interviewer made flexible adjustments to the order and method of asking questions, without imposing any induction or intervention on the participant or judging any of their statements. Each interview was expected to last for 20–30 min. In fact, the interviews lasted for 43–67 min, and each participant was interviewed once.

### 2.5. Data Analysis

The audio recordings were converted into text by the interviewer within 24 h of the interview, and the participant’s movements and expression changes were also interpreted by the interviewer. The data were analyzed using Colaizzi’s 7-step phenomenological method. (1) All the interview data of the 16 participants were carefully and repeatedly read to understand participants’ genuine experiences and feelings about ACS. (2) Important and meaningful statements related to the interview questions were identified and extracted. In this step, 93 meaningful statements about rumination were extracted. (3) Recurring ideas were coded. (4) The encoded ideas were pooled to look for meaningful common concepts to form the prototype themes. In this step, 2 themes and 8 sub-themes were extracted. (5) The participants’ original descriptions were extracted to explain each formed theme in detail. (6) Similar ideas were identified, and formal themes were sublimated. In this study, 2 categories were extracted. (7) The formed themes were returned to the participants for verification to ensure the authenticity of the results. This data analysis process was conducted simultaneously by the first and second authors, respectively, while the corresponding author supervised the process. All the authors engaged in in-depth discussions until a consensus was reached. Finally, the formed themes were returned to the 16 participants for examination to determine whether the themes extracted from the data accurately reflected their feelings and experiences.

## 3. Results

The 16 participants’ demographic and clinical characteristics are shown in Table 1. Based on the interviews with the 16 participants, two themes and eight sub-themes were derived. The two themes were non-adaptive rumination and adaptive rumination. Non-adaptive rumination included four sub-themes: worry or anxiety of daily activities and medical therapy (37.50%), fear of unpredictable outcomes and death (37.50%), sadness of maladjustment (25.00%), and remorse of carelessness (12.50%). Adaptive rumination included four sub-themes: tracing of disease processes (100%), enhancement of disease cognition (81.25%), improvement of health awareness (62.50%), and adjustment of lifestyle cognition (100%) (as shown in Table 2).

### 3.1. Theme 1: Non-Adaptive Rumination

The participants experienced a range of emotional distress in the early stages of ACS. In this theme, four sub-themes were derived: worry or anxiety of daily activities and medical therapy, fear of unpredictable outcomes and death, sadness of maladjustment, and remorse of carelessness.

#### 3.1.1. Sub-Theme 1: Worry or Anxiety of Daily Activities and Medical Therapy

Some patients experienced worry or anxiety due to disease treatment, return to work, family responsibilities, economic pressure, etc.

N2: “I hear from doctors and nurses that I will take aspirin for a long time, and will need to pay attention to bleeding (the brow frowning)”.

N6: “I have learned from the Internet that coronary angiography agents cannot be excreted from the body and can cause fever and death (looking at the researcher anxiously)”.

N4: “I am worried about my wife and children (looking at his wife), and then after discharge, whether I can do my previous job, because I am a physical worker”.

N10: “What I worried about first is whether the disease will delay my teaching schedule in September, because I will go back to class (taking a deep breath)”.

N12: “Now that I am sick, so the family’s financial pressure suddenly becomes very heavy (tears infiltrating the eyes)”.

#### 3.1.2. Sub-Theme 2: Fear of Unpredictable Outcomes and Death

All the patients lacked knowledge about ACS before admission. Some patients had previously disregarded the early symptoms of ACS or did not know the severity of the disease before, so that they were frightened by this fact after admission. Some other participants said they feared a sudden attack of myocardial infarction and death.

N8: “In fact, I had chest pains before, but I didn’t pay attention to it. Now I understand that this disease is really dangerous, and even can be life-threatening (a serious expression on face)”.

N12: “When the chest pained, I did not care about it. But after being in hospital, what I listened to the doctor said made me panic, and now I still feel a little scared”. 

N13: “I once saw in the news that drivers in their 30s, 40s and 50s were died of sudden death, especially while driving, which resulted from heart attacks due to excessive fatigue. I am a driver, and I had chest pains several times while driving. Now I think about it, it is too dangerous (frightened eyes)”.

N11: “I do not know when the disease will attack, so I am afraid of a sudden acute attack and then die”. 

#### 3.1.3. Sub-Theme 3: Sadness of Maladjustment

Some patients experienced sad emotions because they failed to deal well with changes in the way one perceives themself, social roles were threatened, etc. 

N11: “When I was first hospitalized for hypertension, the results of computed tomography and echocardiogram were good and my blood vessels and heart were in good condition. But after discharge, I noticed that I had chest pains and chest tightness, so I guessed it could be coronary artery disease. How can I have been diagnosed with coronary heart disease and hypertension at such a young age? What should I do (sighing and looking out the window)”?

N13: “I used to feel great about my body, but now I am sick so that I cannot do anything and cannot take care of my grandson (eyes are glazed)”.

N14: “I got sick for the first time last June and had a brace fitted. It has been almost a year and a half so far, and I think it will take more than ten years to get sick again, and I cannot be so unfortunate, but the fact is that the second hospitalization come so soon (bitterly smiling)”.

#### 3.1.4. Sub-Theme 4: Remorse of Carelessness

Some patients ignored the early symptoms of ACS and did not seek medical attention at the first sign of chest pain until they were actually hospitalized, and they felt remorse for this action.

N8: “In fact, I had chest pains before, but I didn’t pay attention to it”.

N12: “When the chest pained, I did not care about it”.

### 3.2. Theme 2: Adaptive Rumination

From the onset of the heart attack to stabilization after admission, the participants recalled their traumatic experiences and searched for clues previously related to ACS. Subsequently, they actively searched for knowledge about ACS on the Internet and carefully received health education from doctors and nurses. So they were better informed about the disease and described their health plan after discharge. In this theme, four sub-themes were derived: tracing of disease processes, enhancement of disease cognition, improvement of health awareness, and adjustment of lifestyle cognition.

#### 3.2.1. Sub-Theme 5: Tracing of Disease Processes 

All the participants traced their disease processes. They recalled what happened before and after admission and realized that, in fact, they had experienced early warning symptoms of ACS.

N2: “When I used to ride my bike, the more I rode, the powerful felt. But slowly I realized that I got out of breath easily when I rode uphill and also when I climbed hills. When I had an attack, my chest was uncomfortable, my throat was uncomfortable and tight, and I sweat (pointing to heart and throat)”.

N4: “I had a sudden attack of chest pains at night, and then transferred to the upper abdomen, with a lot of sweating and breathing difficulties, and then I asked my wife to make an emergency call immediately (the right hand gesticulating from the heart to the upper abdomen and frowning)”.

N13: “I had chest pains several times while driving”.

N14: “At the beginning, my chest was pained so that I could not sleep. After about an hour or two hours, I began to sweat”.

#### 3.2.2. Sub-Theme 6: Enhancement of Disease Cognition

The participants said that they knew nothing about ACS or even never heard of it before they became sick, and, after hospitalization, their cognition of ACS had enhanced to varying degrees.

N1: “I didn’t understand before, but now I know that I cannot make myself too tired and do heavy physical work”.

N2: “In the past, I just knew that one person I knew had died of a heart disease but I didn’t know anything about heart disease. When I am in hospital these days, I have learned a Mediterranean diet and low-salt and low-fat diet on the internet”.

N3: “Now I understand that people with irregular lifestyle, a family history of ACS, a heavy-oil and heavy-salt diet, alcohol consumption, and lack of exercise are more likely to develop myocardial infarction, of which the most common symptoms are chest tightness and chest pains”.

#### 3.2.3. Sub-Theme 7: Improvement of Health Awareness

Many patients said that they knew that good health was important before, but they did not care about it. Their health awareness had been greatly improved after their experience of heart disease.

N3: “I used to enjoy drinking beverages, although I knew they were not good to health. But now I know that I must be self-disciplined, and I have a family history of heart disease, so I can not drink beverages anymore”.

N4: “I knew that smoking and drinking were bad to health before, but I always could not quit, and I didn’t take them seriously (smiled ashamedly). I must make up my mind to quit smoking and drinking after discharge. Health is so important, and I must put my health first”.

N6: “Now I will take my health seriously (clenched fist)”.

N14: “When I got sick the first time, I tried to exercise and quit smoking. But half a year later, when I returned to work, I gave up slowly and smoked more and more. But after being discharged this time, I dare not smoke anymore”.

#### 3.2.4. Sub-Theme 8: Adjustment of Lifestyle Cognition

All the participants said that they must change their bad living habits after discharge.

N2: “After discharge, I will keep exercising, live a regular life, not stay up late, and follow nurses’ advice of a low-salt and low-fat diet”.

N3: “Be sure to control my diet and exercise regularly, follow a diet with less oil and salt, drink less beverages, eat more vegetables and fruits, take in the right amount of protein, and keep a good mindset”.

N6: “I think I am so thin that after discharge I have to strengthen exercise to try to avoid a recurrence of heart disease”.

N10: “If I am in a bad mood or stressed, I will let it out instead of being in inner turmoil”.

N14: “After this discharge, I will take my medication regularly and not stop or take less medication without authorization”.

## 4. Discussion

This study used qualitative research methods to investigate the true feelings and experiences and the content and nature of rumination in Chinese middle-aged and young patients with ACS. Two themes were extracted: non-adaptive rumination and adaptive rumination. Non-adaptive rumination included four sub-themes: worry or anxiety of daily activities and medical therapy, fear of unpredictable outcomes and death, sadness of maladjustment, and remorse of carelessness. Adaptive rumination included four sub-themes: tracing of disease processes, enhancement of disease cognition, improvement of health awareness, and adjustment of lifestyle cognition. These findings were similar to those found in patients with cancer [19], elderly female patients with AMI [14], para sport athletes [30], etc. This provides a fundamental understanding of rumination experiences in Chinese young and middle-aged patients with ACS and provides new data for healthcare providers when designing intervention programs to enhance PTG in these patients.

After experiencing ACS, young and middle-aged patients showed negative emotional changes with characteristics of PTSD, similar to the results reported by Meli et al. [31]. There are three possible reasons for this: Firstly, the abruptness of the event, the actual risk of death, and the perceived loss of control and helplessness during the event represent potential trauma [6]. Intrusive thinking also focuses on the fear of future recurrent cardiac events, not just cardiac events that have already occurred [32]. As some of the patients in this study said, they feared a sudden myocardial infarction and then death. Another patient who was hospitalized a second time for ACS said he felt helpless about the second hospitalization. Secondly, young and middle-aged adults, as the mainstay of society and their family, will have to bear the pressure after the diagnosis of ACS in many aspects. In this study, young and middle-aged patients with ACS experienced worry or anxiety, fear, sadness, and remorse due to disease treatment, daily activities, unpredictable outcomes and death, economic pressure, inability to deal with changes in the way they perceives themselves, threatened social roles, the challenge of returning to work, and ignorance of early symptoms of ACS. Thirdly, there was a lack of disease-related knowledge. In this study, all the patients were unaware of knowledge about ACS, which was similar to the results reported by Thanavaro et al. [33]. In interviews, one patient was worried about the long-term use of aspirin after discharge and its side effects, and another was worried about the side effects of the contrast media used in coronary angiography. Some patients ignored the warnings or early symptoms of ACS and suffered from fear and remorse after being admitted to the hospital and learning how dangerous the disease was. A study showed that a lack of, inadequate, or incorrect disease-related knowledge caused patients to felt unprepared and overwhelmed, which often led to feelings of despair, frustration, and anger [34]. However, timely information support can alleviate anxiety, depression, and the psychological impact of the illness [35]. Therefore, it is imperative for medical staff to communicate more with patients to recognize their negative emotions. 

Rumination is a dynamic process. Processes that initially run automatically (e.g., negative thoughts about experiences) are gradually replaced by an awareness of trying to find meaning in traumatic events and integrate these experiences into life [36]. In addition, adaptive rumination will occur repeatedly even in the absence of direct demand, including three dimensions: past, present, and future, which can play an enduringly positive role in the development of individuals’ emotions, behaviors, and events [37]. In this study, although young and middle-aged patients with ACS had negative emotions and lacked disease knowledge in the early stages of the disease, they slowly accepted the fact that they developed the disease, and they gradually obtained disease-related information from the Internet and medical staff. From the obtained information, they traced their disease processes to look for the causes and early warning symptoms of ACS, self-management knowledge, and other positive factors, and then gradually changed the original disease cognition, improved self-health awareness, and adjusted lifestyle cognition. This was similar to the cognitive processing that occurs in para-sport athletes after experiencing a disability life event [30]. Therefore, medical staff should effectively identify the timing of the transition from non-adaptive to adaptive rumination in young and middle-aged patients with ACS and provide patients with health education to help them better transit to adaptive rumination.

Positive cognitive processing is the result of the interaction of multiple factors. The results of this study showed that resilience and information support played important roles in the transformation of negative to positive cognitive processing in Chinese young and middle-aged patients with ACS. In this study, although the patients experienced some negative emotions, they did not overly fall into them but maintained an optimistic attitude, calmed down, and faced the disease and actively learned disease-related knowledge and made positive adjustments. Resilience has been proven to be negatively correlated with non-adaptive rumination and to be positively correlated with adaptive rumination [38]. Computerized mouse-based (gaze) contingent attention training to improve resilience has been proven to reduce non-adaptive rumination effectively [39]. Therefore, medical staff can take interventions to improve patients’ resilience to help them better deal with negative emotions and actively face the disease. It was not difficult to see from the interview data that patients in this study lacked relevant knowledge about ACS, regardless of education level. A study has shown that a lack of knowledge of ACS symptoms can delay decision making, in turn causing pre-hospital delay [40]. Moreover, a high level of disease knowledge was correlated with fewer negative emotions and positive cognition and emotions [41]. Furthermore, the lack of knowledge may also influence the patients’ awareness of risk factors, health behaviors, and adherence to medication therapy [35]. In this study, after the patients accepted the fact that they were diagnosed with ACS, they actively searched for information about ACS online and actively accepted health education from medical staff and then gradually made positive adjustments. A study showed that the intervention of social media platforms that carry informational posts and chat functions facilitated patient information sharing and interactive support [42]. Therefore, healthcare providers can create a social media site or software or WeChat mini program where they can provide patients with disease-related knowledge, such as how to recognize early symptoms, treatment, risk factors, and self-management of ACS, and can also interact with patients, which will build the patients’ confidence in fighting against the disease, improve their optimism level, and help them transit from non-adaptive rumination to adaptive rumination more quickly.

## 5. Limitations

There were some limitations to this study. Firstly, this study was a short-term study, and the long-term experiences of the participants will be a valuable avenue for future exploration. Secondly, the participants were all recruited from the same hospital. Therefore, multicenter and multistage qualitative studies are required to further investigate the content and nature of rumination in Chinese young and middle-aged patients with ACS in the future. Thirdly, this study was designed for young and middle-aged patients aged 18–59 years, due to the low incidence of ACS in the population under 35 years old, we were unable to include patients with ACS under 35 years old at the research site during our research period, which may cause bias in the results. More representative participants should be recruited to eliminate potential bias in future studies.

## 6. Conclusions

Although Chinese young and middle-aged patients with ACS experience negative emotions after a traumatic cardiac event, they gradually make positive changes. Optimism and information support play important roles in the transformation. Therefore, health providers should actively communicate with patients, recognize their non-adaptive rumination, and provide targeted interventions in a timely manner to facilitate the transformation to adaptive rumination to promote PTG.

## Figures and Tables

**Table 1:** Participants’ demographic and clinical characteristics.

Participants	Gender	Age	Education	Occupation Situation	Occupation Type	Medical Form	Economic Pressures	Comorbidity	Duration since Diagnosis
N1	Female	57	Junior middle school	Employed	Physical labor	Medical insurance	Relatively large	Diabetes	≤Half a year
N2	Male	40	Senior middle school	Employed	Non-physical labor	Medical insurance	A little bit large	Hypertension	Half a year–5 years
N3	Male	35	Junior college	Employed	Non-physical labor	Self-paying	Commonly large	Hypercholesterolemia	≤Half a year
N4	Male	46	Senior middle school	Employed	Physical labor	Medical insurance	A little bit large	Hypertension	≤Half a year
N5	Male	43	Undergraduate college	Employed	Non-physical labor	Medical insurance	Commonly large	Hypertension	≤Half a year
N6	Male	58	Senior middle school	Employed	Non-physical labor	Medical insurance	No	Hypertension	≤Half a year
N7	Male	42	Technical secondary school	Employed	Non-physical labor	Medical insurance	No	Hypertension	≤Half a year
N8	Male	51	Junior middle school	Employed	Physical labor	Medical insurance	A little bit large	Diabetes, hypertension	≤Half a year
N9	Male	59	Junior middle school	Employed	Non-physical labor	Medical insurance	No	Hypertension	≤Half a year
N10	Male	37	Doctoral degree	Employed	Non-physical labor	Self-paying	No	Hypertension, hypercholesterolemia	≤Half a year
N11	Male	47	Master’s degree	Employed	Non-physical labor	Self-paying	Extremely large	No	≤Half a year
N12	Male	57	Primary school	Employed	Physical labor	Medical insurance	A little bit large	Diabetes	≤Half a year
N13	Male	50	Junior middle school	Employed	Non-physical labor	Self-paying	Extremely large	No	≤Half a year
N14	Male	42	Junior middle school	Unemployed	Physical labor	Medical insurance	Commonly large	No	Half a year–5 years
N15	Female	36	Undergraduate college	Employed	Non-physical labor	Medical insurance	A little bit large	No	≤Half a year
N16	Female	47	Technical secondary school	Employed	Non-physical labor	Medical insurance	Commonly large	Hypertension	≤Half a year

**Table 2:** The results of themes and sub-themes.

Themes	Sub-Themes	N (%)
Non-adaptive rumination	Worry or anxiety of daily activities and medical therapy	6 (37.50%)
	Fear of unpredictable outcomes and death	6 (37.50%)
	Sadness of maladjustment	4 (25.00%)
	Remorse of carelessness	2 (12.50%)
Adaptive rumination	Tracking of disease processes	16 (100.00%)
	Enhancement of disease cognition	13 (81.25%)
	Improvement of health awareness	10 (62.50%)
	Adjustment of lifestyle cognition	16 (100.00%)

## Data Availability

The data presented in this study are available on request from the corresponding author due to restrictions of privacy and ethical reasons.

## Supplementary Materials

The following supporting information can be downloaded at: https://www.mdpi.com/article/10.3390/healthcare12161651/s1, Interview guide.
