# Supplementary material for: The Content and Nature of Rumination in Chinese Young and Middle-Aged Patients with Acute Coronary Syndrome: A Qualitative Study
Source: Healthcare (Basel). 2024 Aug 19;12(16):1651. doi: 10.3390/healthcare12161651 (PMC11353411; doi:10.3390/healthcare12161651)
Supplement: Supplementary file 1 [file healthcare-12-01651-s001.zip › healthcare-3098484-supplementary.pdf]

Supplementary Materials

Interview guide:

(1) Before the disease, what was your perception of the disease?

(2) How did you feel at the beginning of the disease?

(3) How did the disease affect you?

(4) What were you most worried about?

(5) After the disease, what was your perception of the disease?

(6) How did you look forward to your future life through this experience?

(7) What changes would you make to your life after discharge?
